# Supplementary material for: End-to-end topographic networks as models of cortical map formation and human visual behaviour
Source: Nat Hum Behav. 2025 Jun 6;9(9):1975–91. doi: 10.1038/s41562-025-02220-7 (PMC12454150; doi:10.1038/s41562-025-02220-7)
Supplement: Supplementary file 1 — Supplementary Tables 1–4 and Figs. 1–26. [file 41562_2025_2220_MOESM1_ESM.pdf]

nature human behaviour

Supplementary information

https://doi.org/10.1038/s41562-025-02220-7

End-to-end topographic networks as models of cortical map formation and human visual behaviour

In the format provided by the authors and unedited

	CNNs		All-TNNs and LCNs		All networks
**Layer**	**Output Shape**	**Parameter #**	**Output Shape**	**Parameter #**	**Filters, kernel size, strides**
Input	(500, 150, 150, 3)	0	500, 150, 150, 3)	0	
**Layer 1**	(500, 48, 48, 64)	9,472	(500, 48, 48, 64)	21,823,488	64, 7, 3
Layer Normalisation	(500, 48, 48, 64)	128	(500, 48, 48, 64)	128	
Max Pooling	(500, 24, 24, 64)	0	(500, 24, 24, 64)	0	
**Layer 2**	(500, 22, 22, 81)	46,737	(500, 22, 22, 81)	22,620,708	81, 3, 1
Layer Normalisation	(500, 22, 22, 81)	162	(500, 22, 22, 81)	162	
**Layer 3**	(500, 20, 20, 81)	59,130	(500, 20, 20, 81)	23,652,000	81, 3, 1
Layer Normalisation	(500, 20, 20, 81)	162	(500, 20, 20, 81)	162	
Max Pooling	(500, 10, 10, 81)	0	(500, 10, 10, 81)	0	
**Layer 4**	(500, 8, 8, 256)	186,880	(500, 8, 8, 256)	11,960,320	256, 3, 1
Layer Normalisation	(500, 8, 8, 256)	512	(500, 8, 8, 256)	512	
**Layer 5**	(500, 6, 6, 256)	590,080	(500, 6, 6, 256)	21,242,880	256, 3, 1
Layer Normalisation	(500, 6, 6, 256)	512	(500, 6, 6, 256)	512	
Max Pooling	(500, 3, 3, 256)	0	(500, 3, 3, 256)	0	
**Layer 6**	(500, 1, 1, 2500)	5,762,500	(500, 1, 1, 2500)	5,762,500	2500, 3, 1
Layer Normalisation	(500, 1, 1, 2500)	5,000	(500, 1, 1, 2500)	5,000	
Flatten	(500, 2500)	0	(500, 2500)	0	
Readout Dense	(500, 565)	1,413,065	(500, 565)	1,413,065	
Output Activation	(500, 565)	0	(500, 565)	0	

Table S1 | Overview of architectural details and parameters of each network component for All-TNNs, locally connected networks, and convolutional networks.

Model	Early stopping epochs
CNNs	35, 35, 35, 35, 35
LCNs	35, 35, 35, 35, 35
All-TNNs ($\alpha = 1$)	35, 35, 35, 35, 35
All-TNNs ($\alpha = 10$)	300, 270, 260, 300, 230
All-TNNs ($\alpha = 100$)	600, 600, 600, 600, 600
All-TNNs SimCLR ($\alpha = 10$)	600, 600, 600, 600, 600
All-TNNs SimCLR finetuning	600, 600, 600, 600, 600
All-TNN shifted ($\alpha = 10$)	300
All-TNN 8 neighbourhood ($\alpha = 10$)	300

Table S2 | Training epochs used for early stopping for all model instances. Epochs were selected by choosing either the epoch during training in which the validation cross-entropy loss started increasing or the 600th epoch.

Model	Mean accuracy map agreement (Pearson correlation)	95% CI	Group-level Noise ceiling
CNN	0.096	(0.095, 0.098)	0.869
LCN	0.235	(0.234, 0.237)	
All-TNN (α=1)	0.292	(0.291, 0.294)	
All-TNN (α=10)	0.306	(0.304, 0.308)	
All-TNN (α=100)	**0.333**	(0.331, 0.335)	
SimCLR All-TNN (α=10)	0.271	(0.269, 0.273)	
TDANN supervised	0.085	(0.083, 0.086)	
TDANN self-supervised	-0.077	(-0.079, -0.075)	
Shifted All-TNN (α=10)	-0.124	(-0.123, -0.125)	

Table S3 | Model agreement with human spatial biases in visual behaviour. Model agreement with humans is computed by the average Pearson correlation between human accuracy maps (averaged across participants) and accuracy maps of each model. Group-level human noise ceiling was calculated using split-halves with Spearman-Brown correction. All models were trained on the ecoset dataset, except for the TDANN models (both supervised and self-supervised), which were trained by the original authors on imagenet (ILSVRC). All models are trained using 5 seeds, with the exception of the TDANN supervised model and the Shifted All-TNN model (α=10), which are trained using one seed.

Model	Mean ADM agreement (Spearman correlation)	95% CI	Noise ceiling
CNN	0.020	(0.009, 0.031)	0.667
LCN	0.081	(0.072, 0.091)	
All-TNN (α=1)	0.070	(0.057, 0.083)	
All-TNN (α=10)	**0.185**	(0.170, 0.199)	
All-TNN (α=100)	0.153	(0.134, 0.173)	
SimCLR All-TNN (α=10)	-0.014	(-0.029, 0.001)	
TDANN supervised	-0.002	(-0.012, 0.008)	
TDANN self-supervised	0.112	(0.098, 0.125)	
Shifted All-TNN (α=10)	0.008	(0.027, -0.012)	

Table S4 | Model agreement with human object-specific biases in visual behaviour. Model ADM agreement is computed by Spearman correlation between the average human ADM and the ADMs of models. Group-level human noise ceiling was calculated using split-halves with Spearman-Brown correction. All models were trained on the ecoset dataset, except for the TDANN models (both supervised and self-supervised), which were trained by the original authors on imagenet (ILSVRC). All models are trained using 5 seeds, with the exception of the TDANN supervised model and the Shifted All-TNN model (α=10), which are trained using one seed.

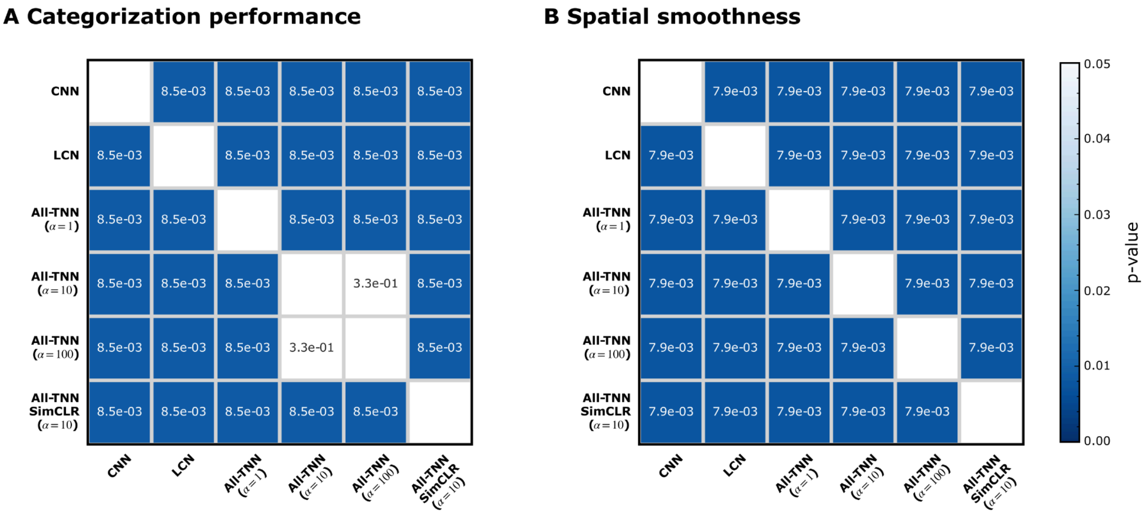

Figure S1 | Statistical test comparing the classification performance and weight smoothness of all models. ***A.*** *BH-FDR-corrected p-values resulting from two-sided permutation test (n=1e4) between classification accuracy on the ecoset test set of pairs of models.* ***B.*** *BH-FDR-corrected p-values resulting from two-sided permutation test (n=1e4) between spatial weight smoothness of pairs of models after training.*

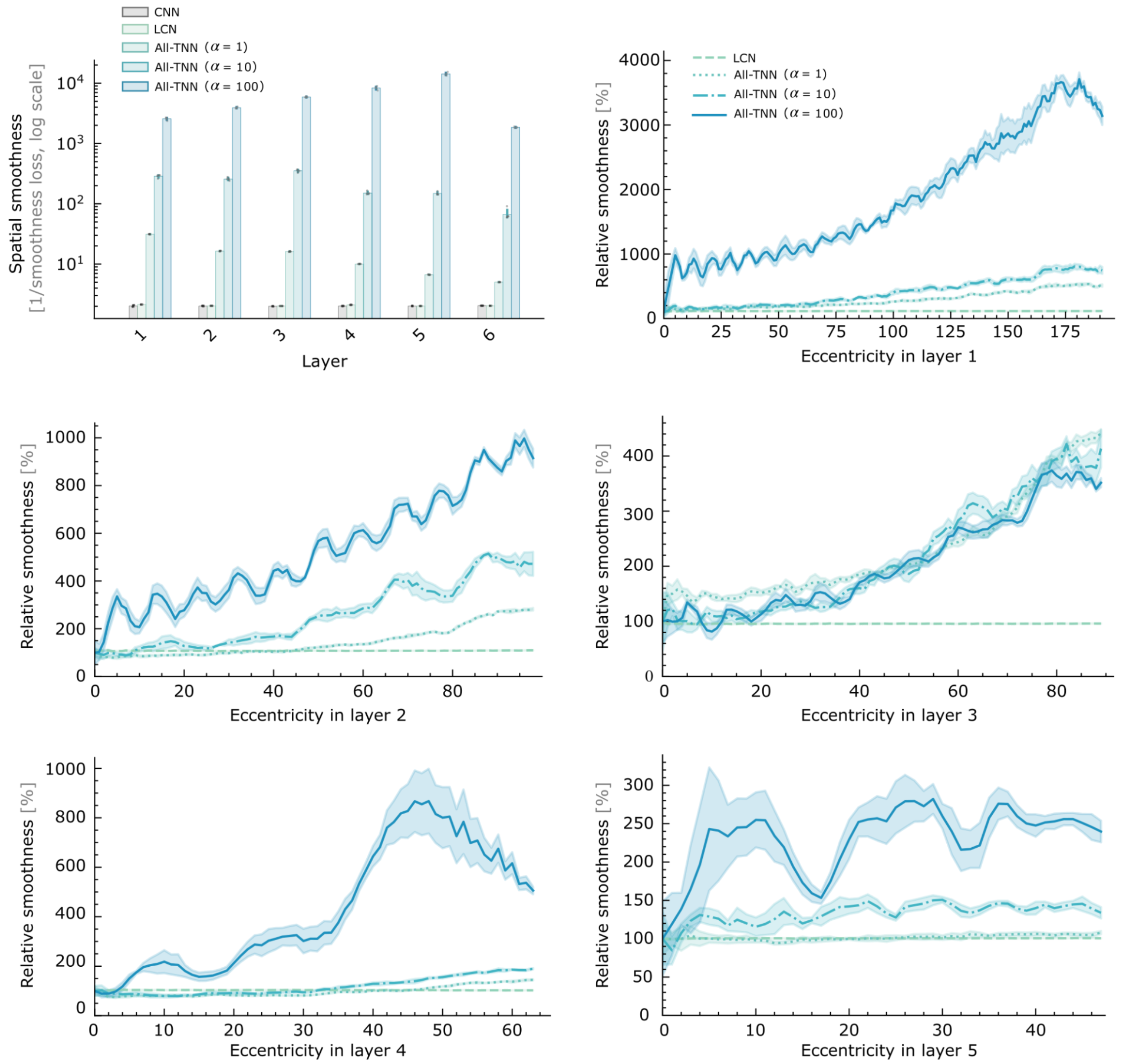

Figure S2 | Global weight smoothness across all model layers. *Top left: cumulative smoothness between neighbouring weights within each layer of all main trained models (see Methods, 5 seeds per model). All other panels depict the weight smoothness relative to the centre unit per eccentricity from the centre unit of each layer for all models.*

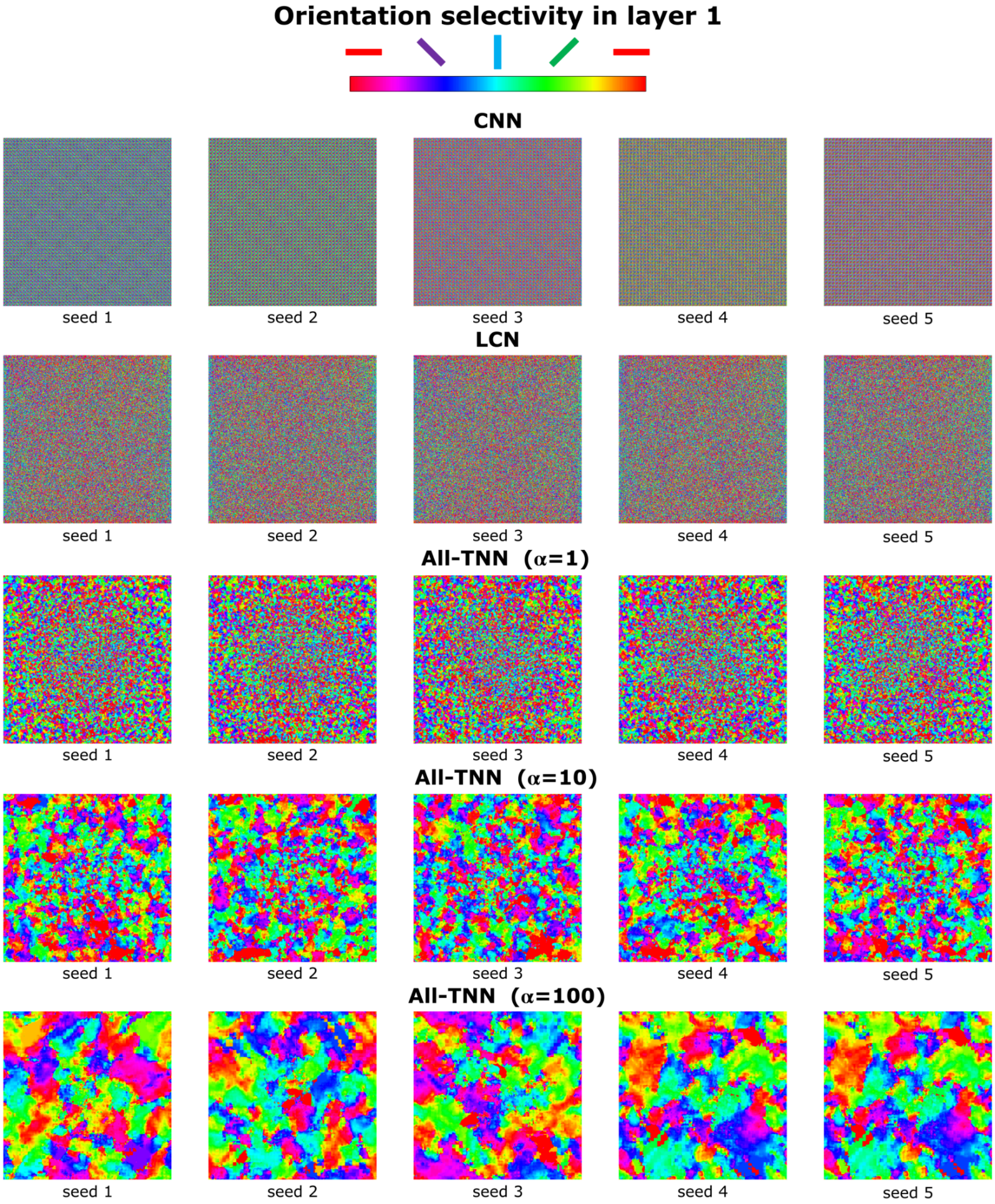

Figure S3 | Orientation selectivity maps for all model instances. *The organisation of orientation selectivities for All-TNNs is consistently smooth across all five network seeds. All trained CNN and LCN seeds show salt-and-pepper orientation selectivity maps. Maps are shown for the early stopping epoch of each model (see Supp. Table 2).*

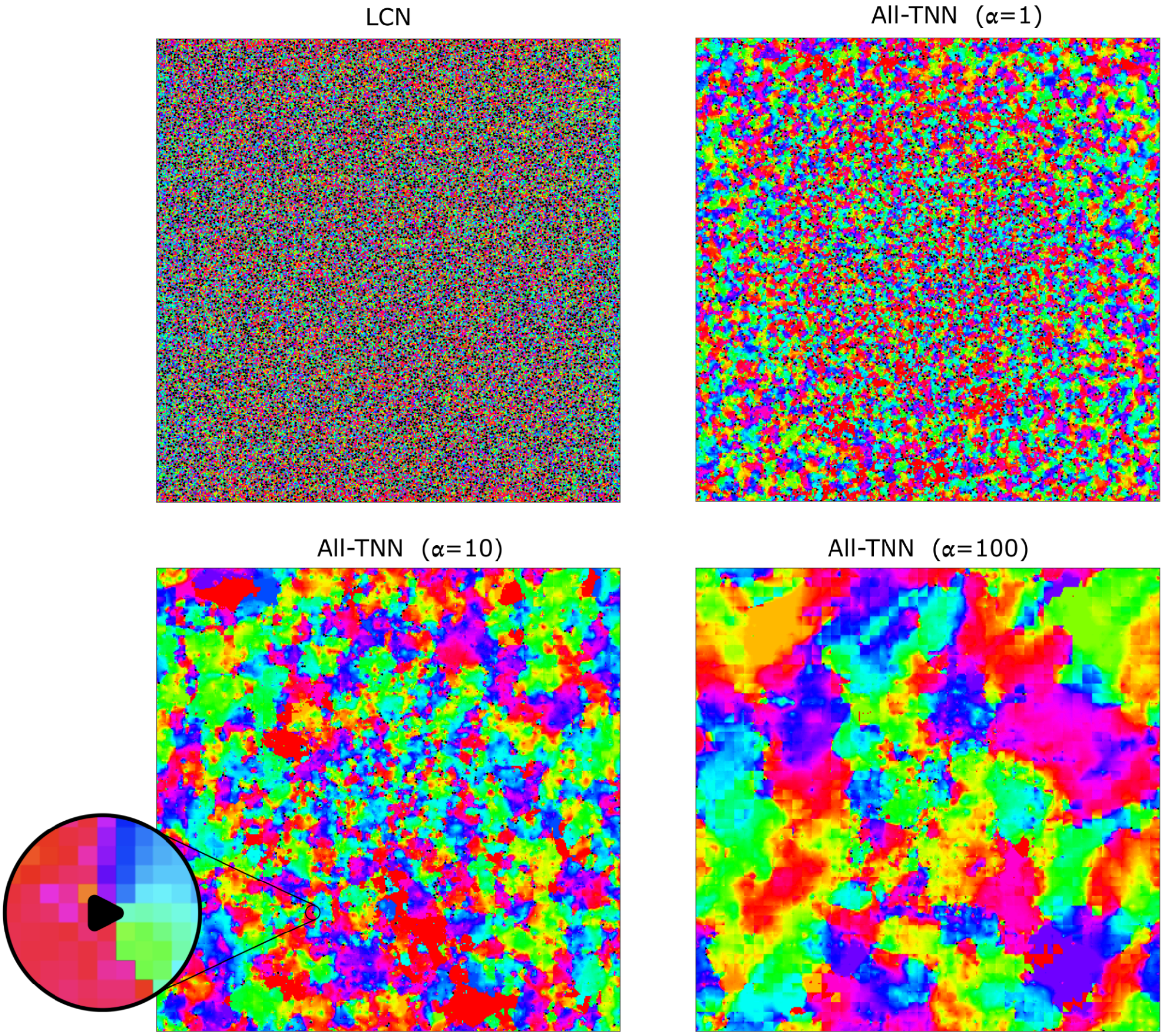

Figure S4 | Pinwheel singularities. *We quantified pinwheel discontinuities (shown as black marks in the plots) in orientation selectivity maps in the first layer of our networks (seed 1). Locally connected networks have salt-and-pepper orientation selectivity maps, which causes many pinwheels to be detected, as discontinuities where all orientation selectivities meet are abundant. In All-TNNs, we observe more V1-like smooth orientation selectivity maps instead of salt and pepper maps. With increasing magnitudes of smoothness loss (α) maps become smoother, and, accordingly, the number of pinwheels decreases.*

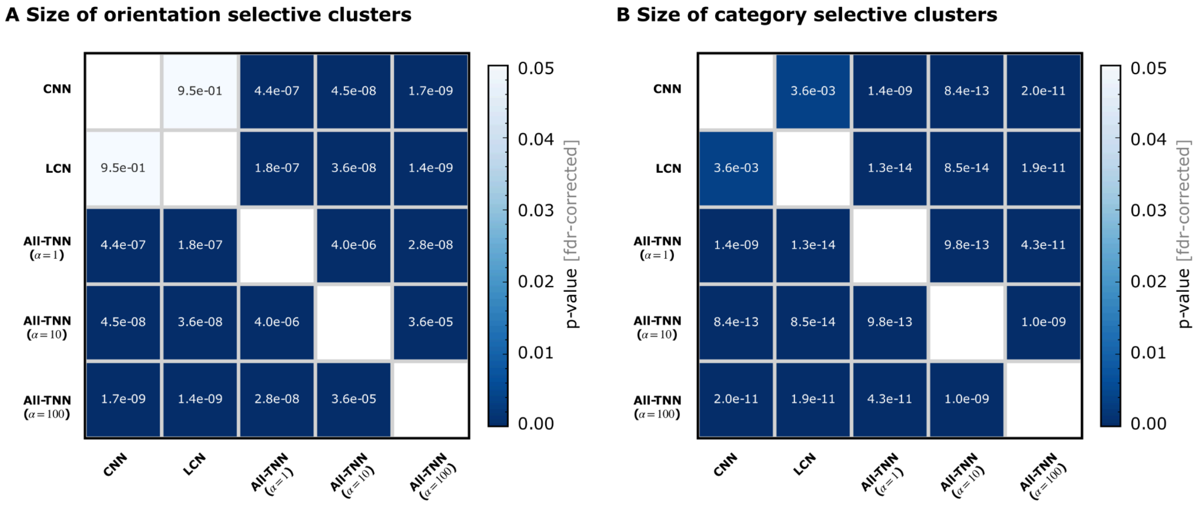

Figure S5 | Statistical test comparing orientation selective cluster sizes of all models. *A.* *BH-FDR-corrected p-values resulting from independent two-sided t-tests between pairs of models for orientation selective clusters in the first layer.* ***B.*** *BH-FDR-corrected p-values resulting from independent two-sided t-tests between pairs of models for category selective clusters in the final layer.*

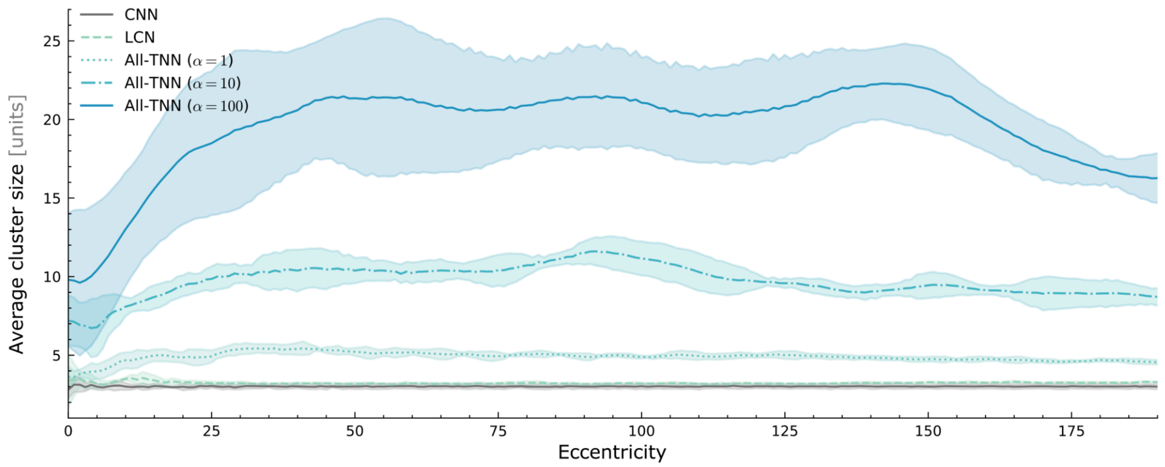

Figure S6 | Radial cluster size of orientation selective units. *Average cluster size per eccentricity from the centre unit of the first layer of each model. The shaded region indicates the 95% confidence interval.*

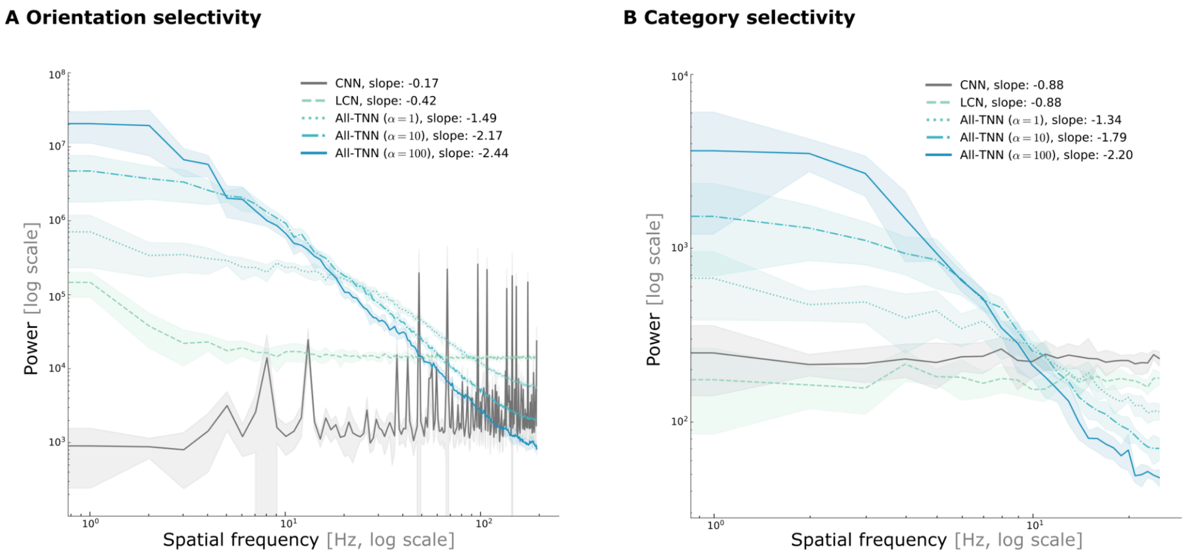

Figure S7 | Smoothness of orientation and category selectivity maps. ***A.*** *Average power spectrum of orientation selectivity in the first layer of all main models.* ***B.*** *Average power spectrum of category selectivity in the last layer. Shaded areas indicated the 95% confidence interval.*

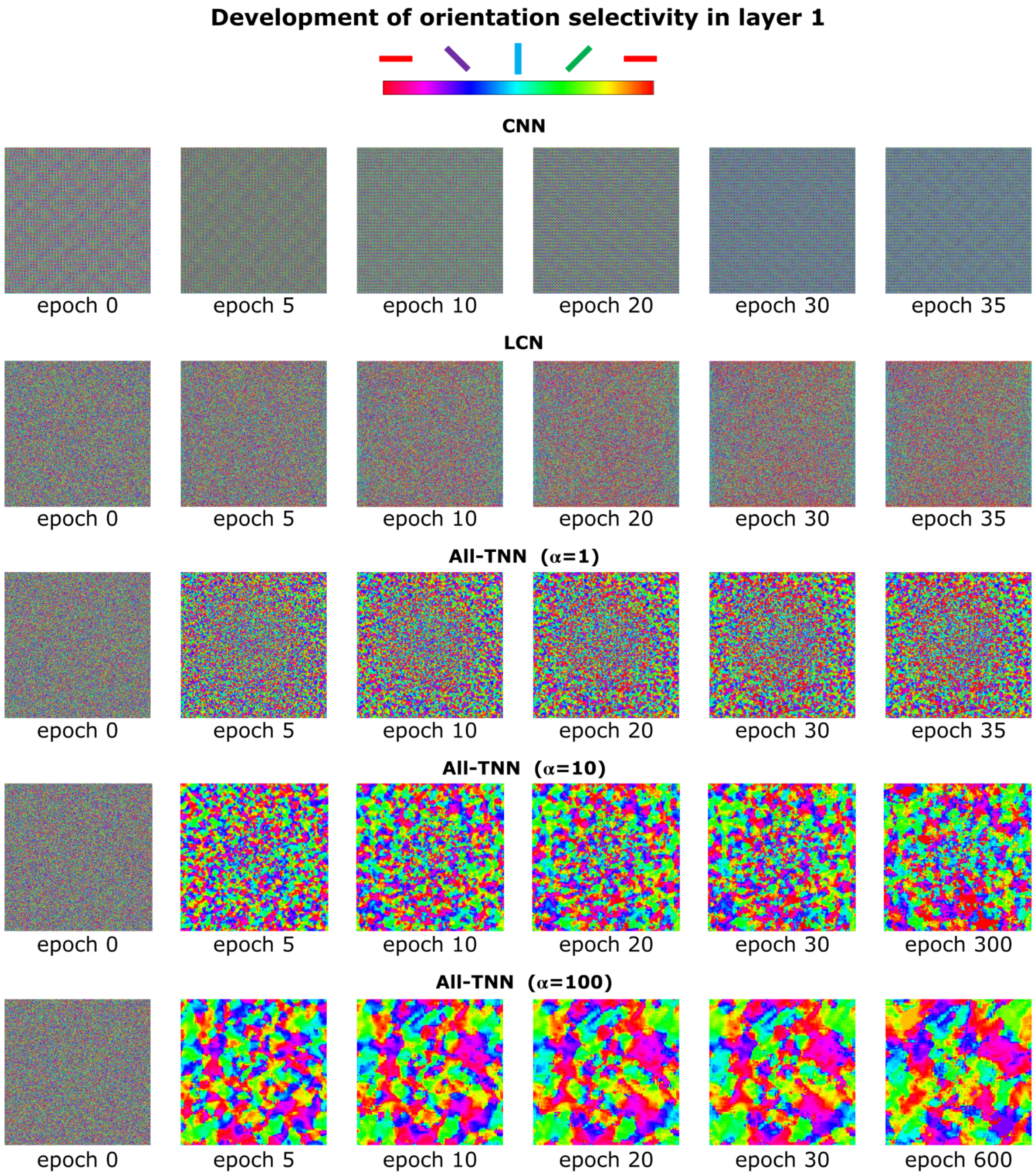

Figure S8 | Orientation selectivity maps across training epochs. *The maps shown are up until the early stopping epoch of each model (see Supp. Table 2). The organisation of orientation selectivities in the first layer of All-TNNs ($\alpha \in [1, 10, 100]$) remains stable after emergence in the first training epochs.*

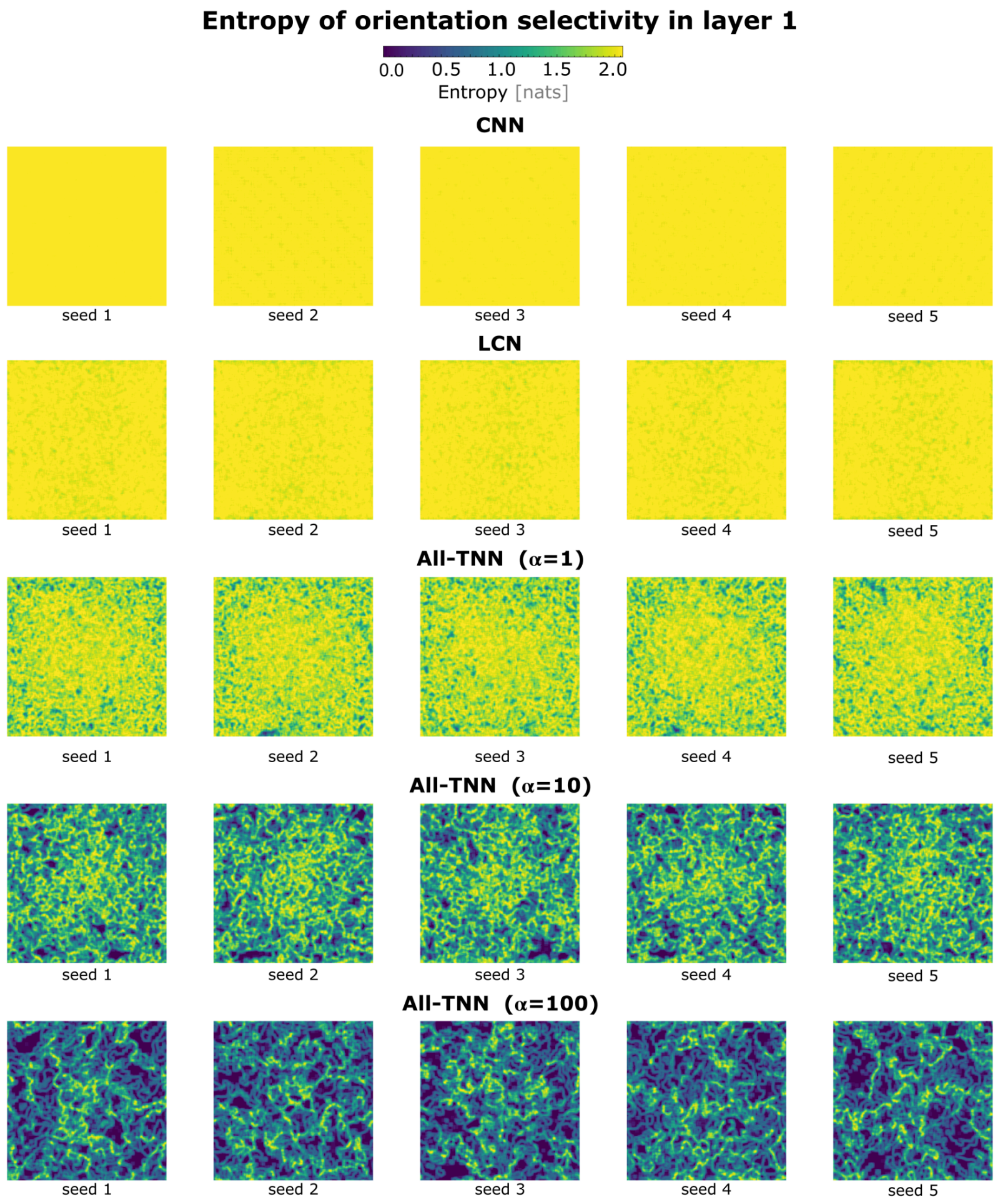

Figure S9 | Entropy analysis across model instances. *Entropy maps of the orientation selectivity in the first layer of All-TNNs ($\alpha \in [1, 10, 100]$) show diverse feature selectivity consistently across network seeds. In contrast, homogenous entropy maps emerge in all trained LCN and CNN seeds.*

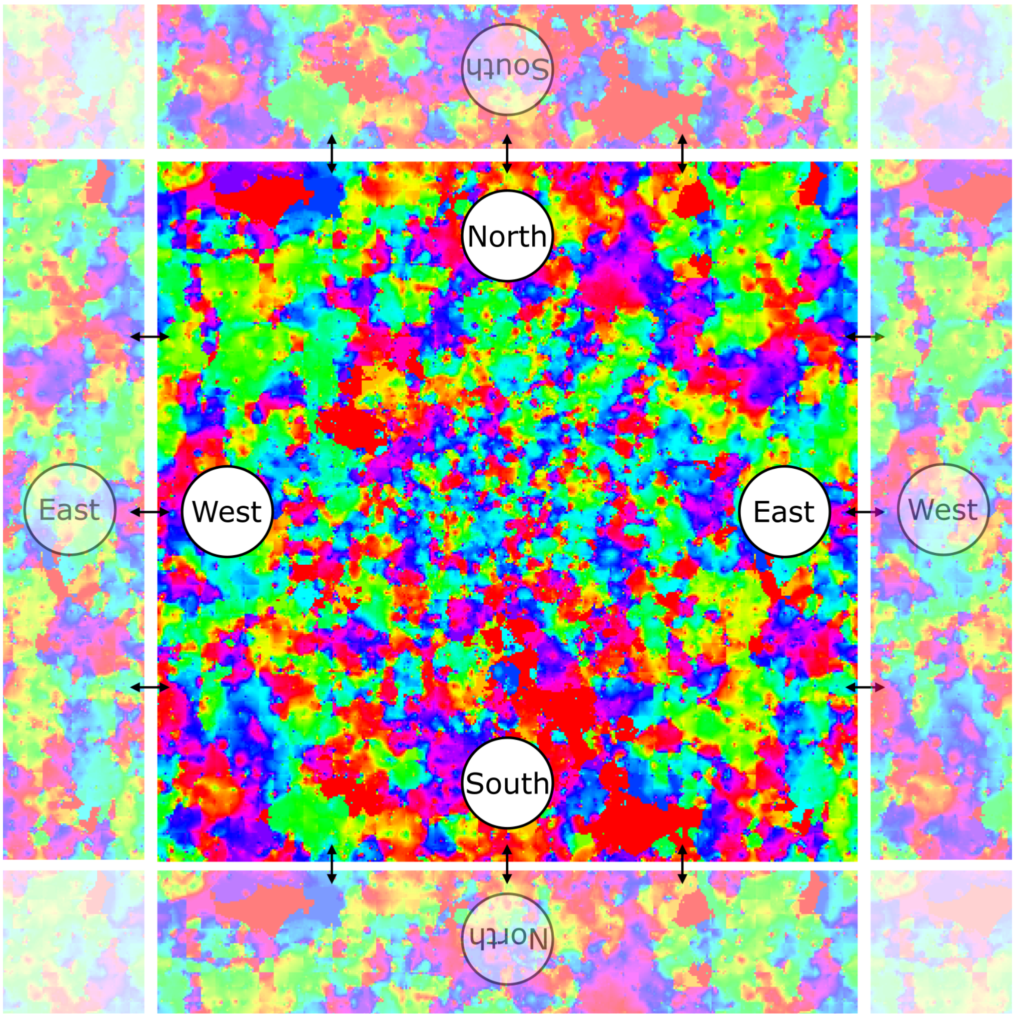

Figure S10 | Smoothness loss is applied in toroidal fashion in All-TNNs. *To avoid border effects that may arise if units on the edge of the sheet are less subject to the smoothness loss because they have fewer neighbours, the smoothness loss is applied in a toroidal fashion. That is, units at each edge of the map are driven to be similar to units at the opposite edge. To illustrate the impact of applying the loss in this toroidal fashion, the figure shows the orientation selectivity map of the first layer of an All-TNN ($\alpha = 10$, seed 1, epoch 300) , where the 'eastern' border is copied over to align with the 'western' border, etc. (illustrated by black arrows). This shows that, indeed, units at one edge of the sheet share similar selectivities with units at the opposite border.*

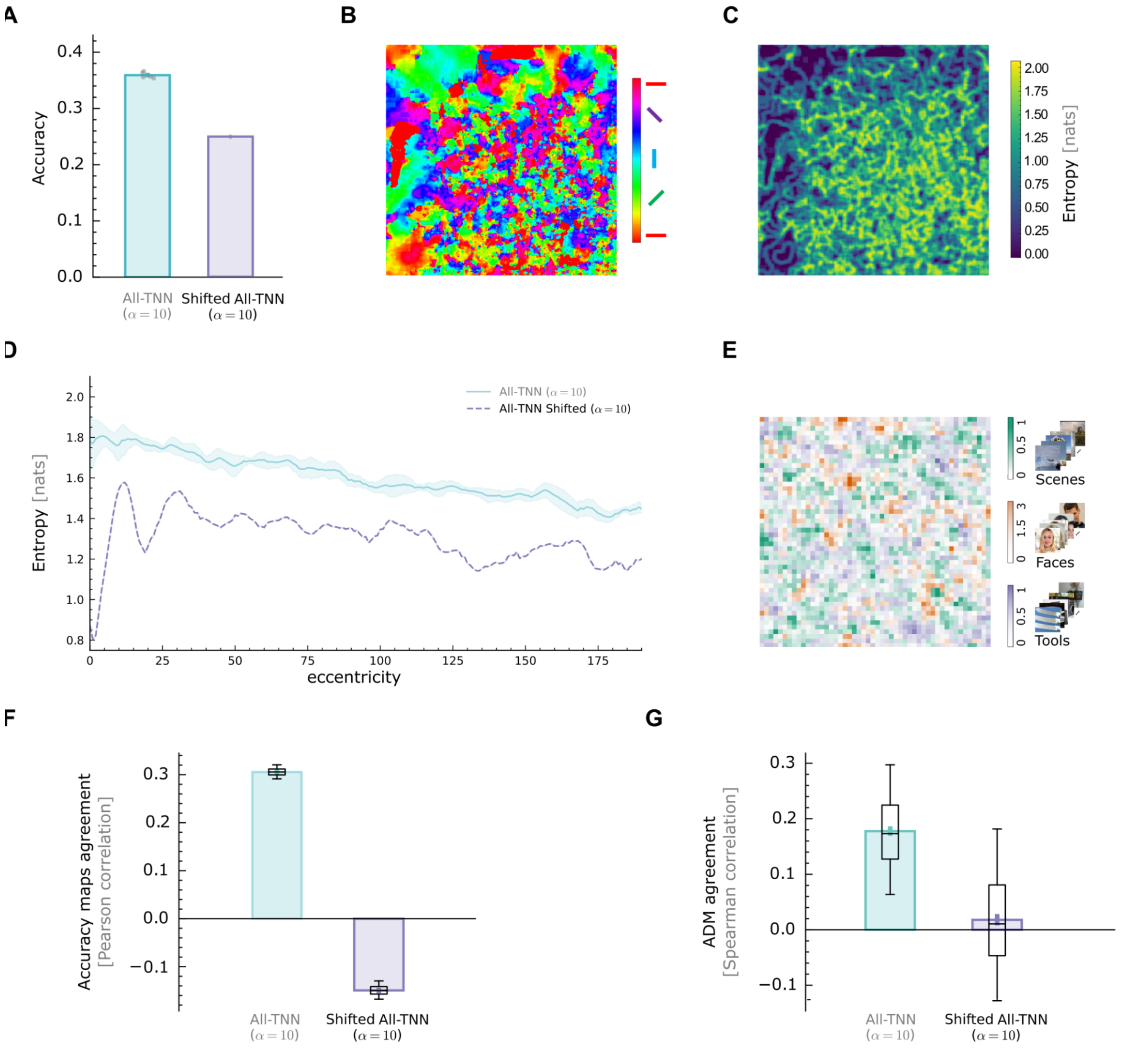

Figure S11 | All-TNN trained on a shifted dataset. *To confirm that the topographical organisation of All-TNNs emerges due to task-relevant dataset statistics, we train one model instance of All-TNN ($\alpha = 10$) on the ecoset training dataset that is augmented by shifting the images 30 pixels to the bottom right.* ***A.*** *The model (one seed) achieves worse classification accuracy than regular All-TNNs ($\alpha = 10$, n=5; data are presented as mean values with 95% confidence interval).* ***B.*** *Orientation selectivity maps emerge.* ***C.*** *The central region with high feature variety (measure using Shannon entropy on the orientation selectivity) that was observed in regularly trained All-TNNs is shifted to the bottom right for the model trained on a shifted dataset, indicating that this region follows dataset statistics.* ***D.*** *The qualitative observation in c is confirmed quantitatively: the high entropy region decreases with eccentricity from the 'centre', which we have shifted by 30 pixels.* ***F.*** *The model does not align well with bootstrapped average human accuracy maps resulting from the behavioural experiment (30 participants, 1 seed for the model, 5 seeds for regular All-TNN; data are presented as mean values with 95% confidence interval).* ***G.*** *The model ADMs do not correlate as well with human ADMs as is the case for regularly trained All-TNNs (30 participants, 1 seed for the model, 5 seeds for regular All-TNN; data are presented as mean values with 95% confidence interval). For both F and G, boxplots show the data distribution, midline of the box represents the median, box limits indicate the 25th to the 75th percentile and whiskers extend to the 5th and 95th percentiles.*

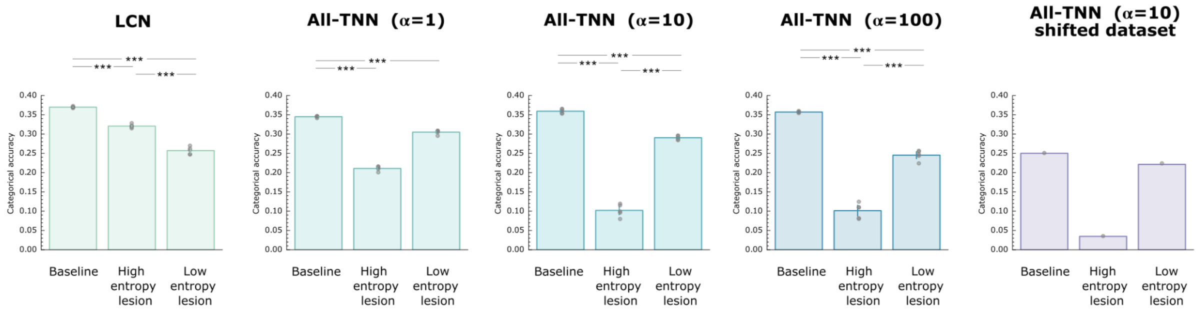

Figure S12 | All-TNNs are more robust to lesions in low entropy regions than in high entropy regions. *Lesioning 25% of randomly selected units in high or low entropy regions and measuring performance on the test set reveals that All-TNNs are more harmed by high entropy lesions with increasing smoothness regularisation (1 seed for the shifted model, 5 seeds for all other models; data are presented as mean values with 95% confidence interval). Statistical testing done with dependent two-sample t-test (two-sided) between conditions, BH-FDR-corrected.*

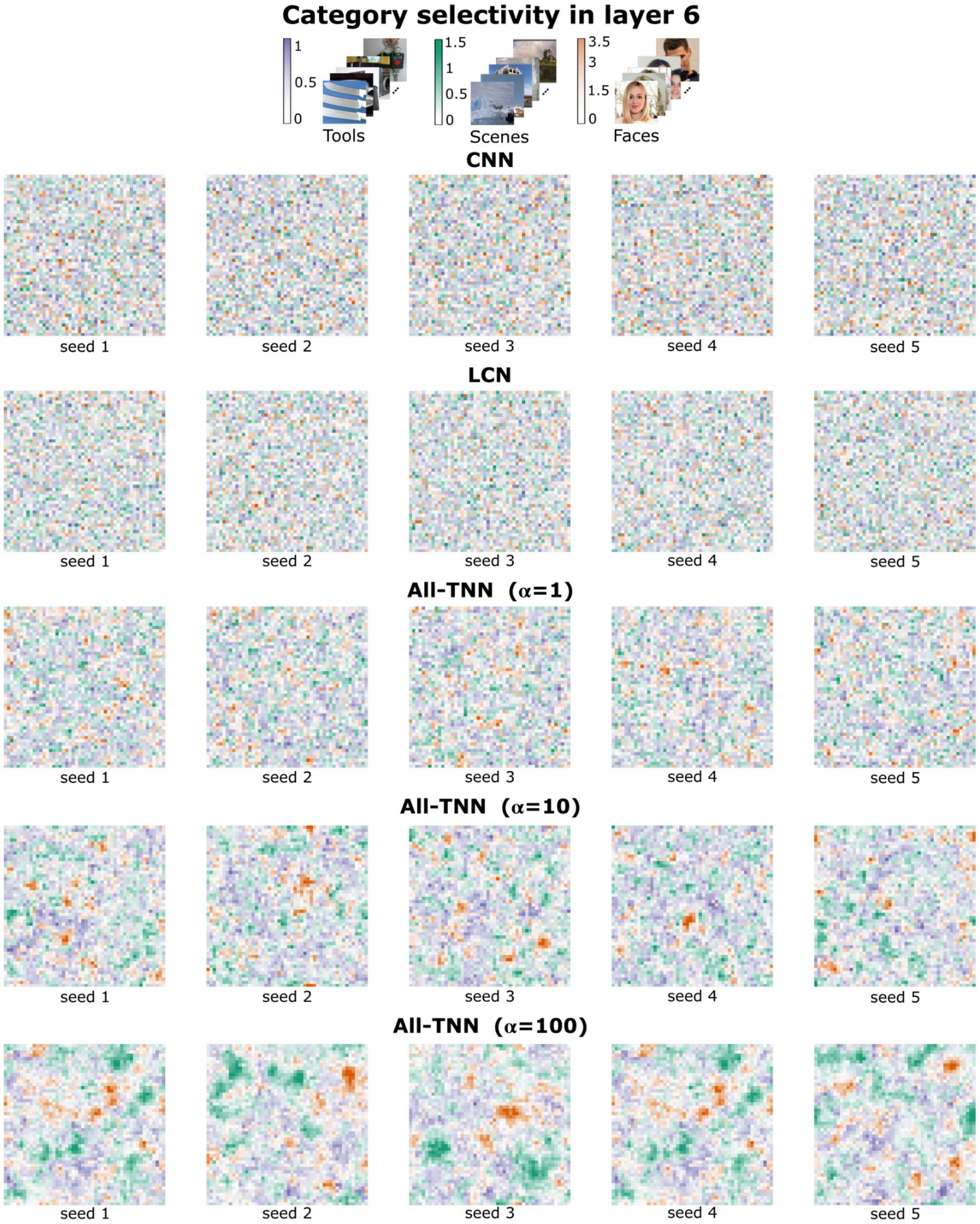

Figure S13 | Category selectivity maps across model instances. *The emergence of clusters of category selectivity for All-TNNs ($\alpha \in [1, 10, 100]$) is consistent across all trained network seeds. Category selectivity maps are unstructured in all trained LCN and CNN seeds. Maps shown for the early stopping epoch of each model seed (see Supp. Table 2).*

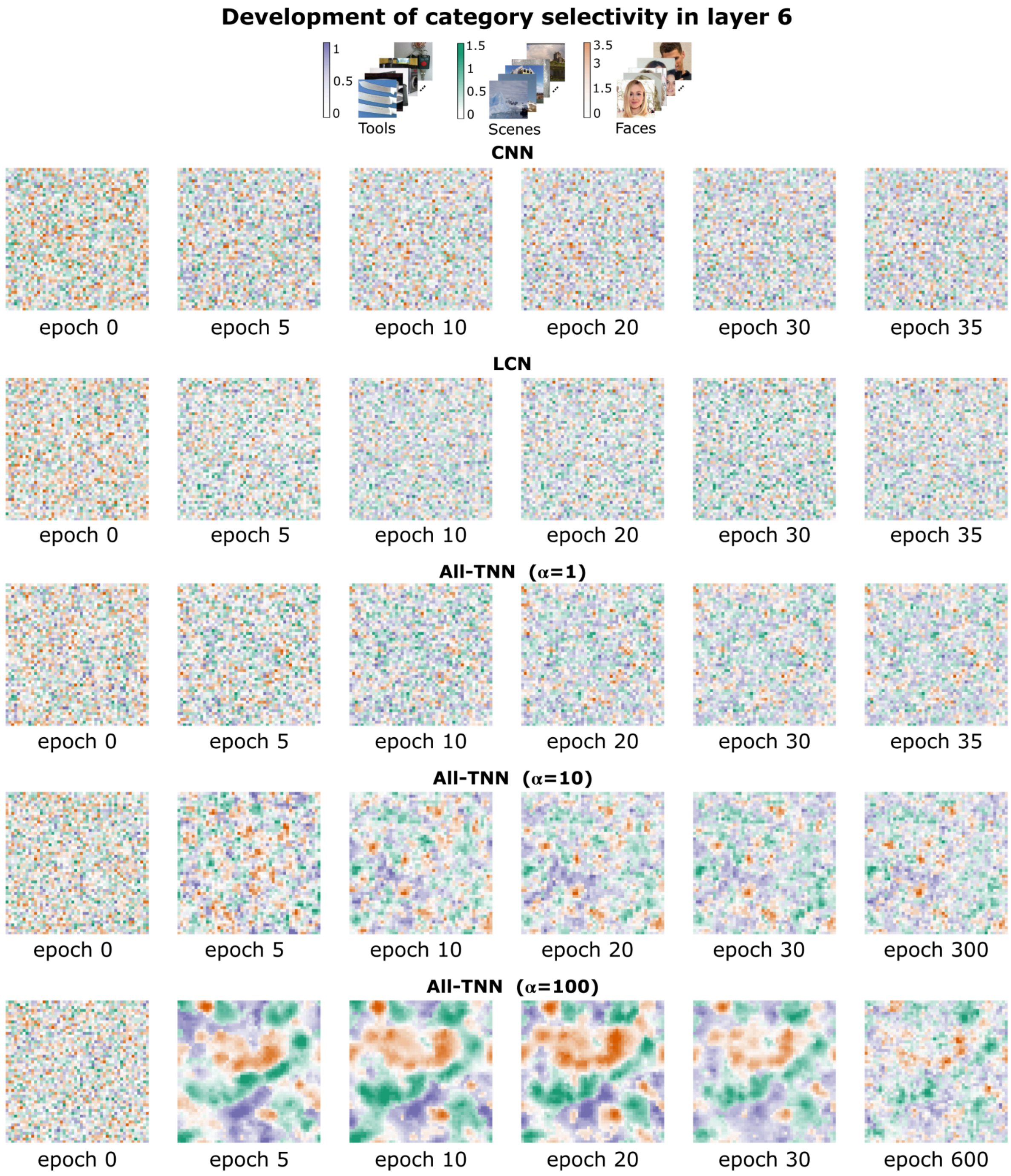

Figure S14 | Category selectivity maps across training epochs. *The clustering of high-level category-based selectivities (d') for tools, scenes, and faces in the last layer of All-TNNs emerges through training epochs. Maps shown up until the early stopping epoch for each model (see Supp. Table 2).*

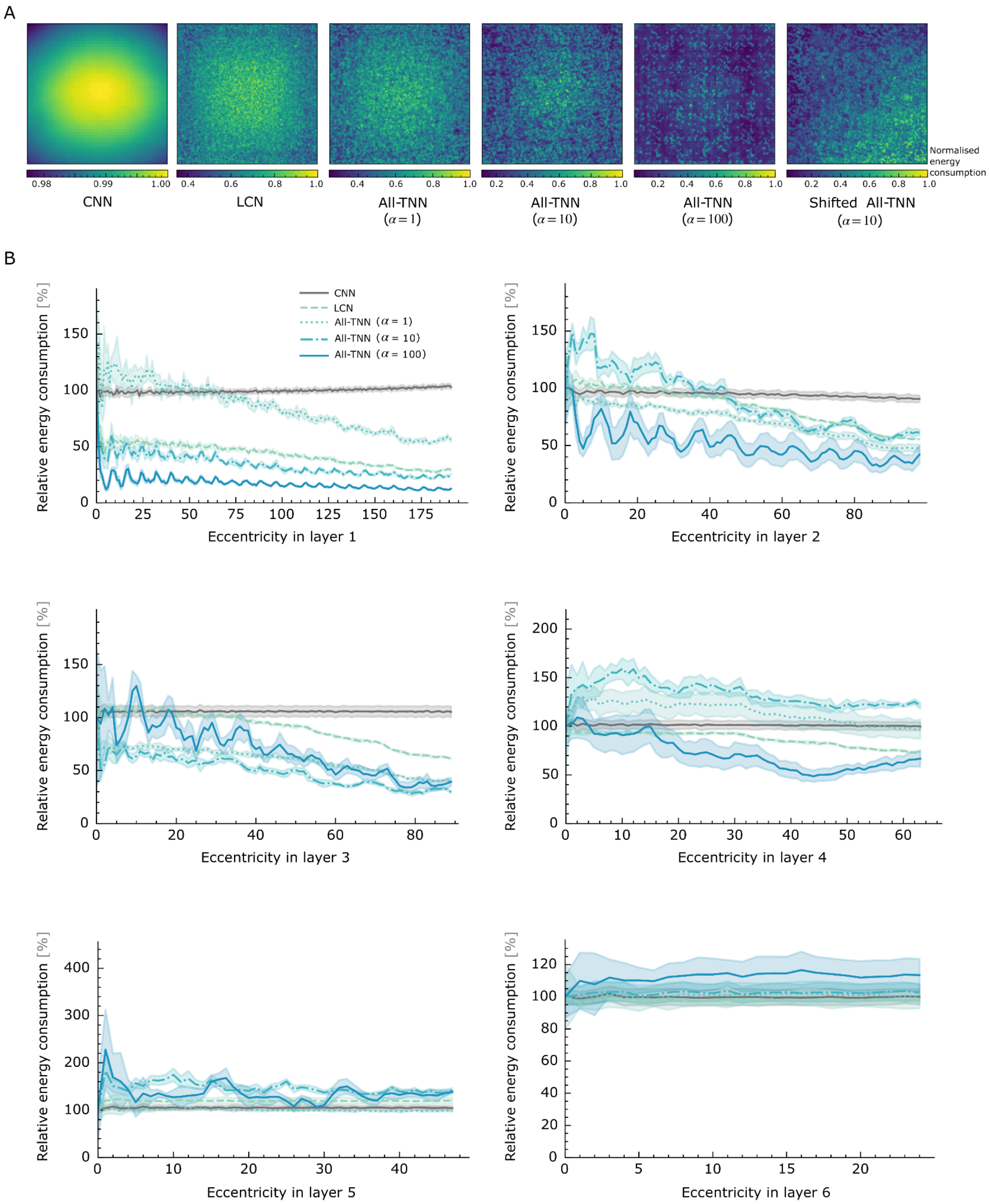

Figure S15 | Energy consumption distributions for all models. ***A.*** *In order to see small variations in the energy consumption maps that may have been invisible in Fig. 3 due to the fact that all models share the same colorbar, we show energy consumption maps where each model map has its individual colorbar. As in Fig. 3, each panel shows the energy consumption for each model (averaged over seeds), averaged across layers 1-5. Unlike in Fig. 3, the CNN panel here shows energy consumption averaged over channels instead of unfolding the layer in the way we typically do throughout the paper, to show the energy consumption in the native arrangement of the CNN. This shows that CNNs have a very marginal drop in energy consumption in the periphery (2%), which is much smaller than LCNs (~60%) and All-TNNs (e.g. >80% for α=10).* ***B.*** *Depiction of the change in*

energy consumption relative to the centre unit in each layer (data averaged across all seeds, shaded region indicates the 95% confidence interval).

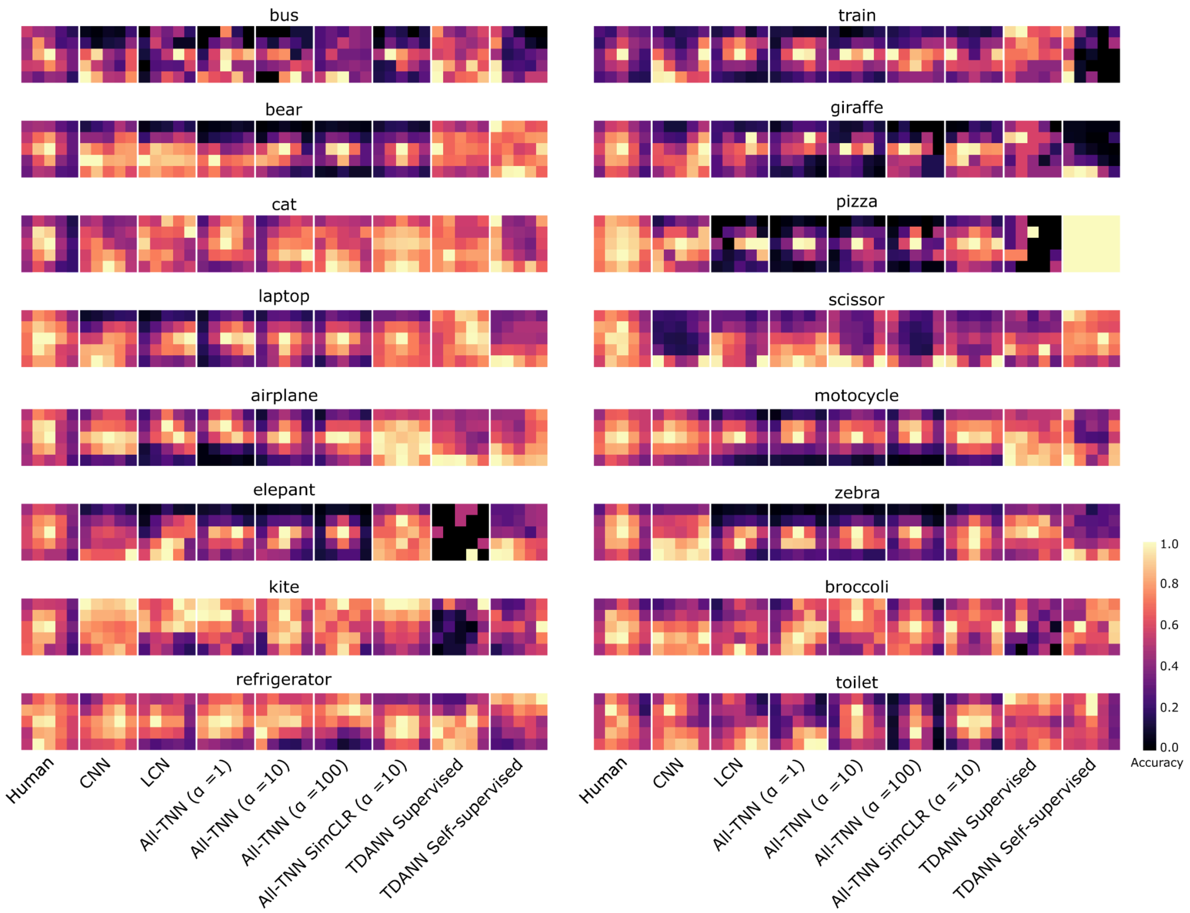

Figure S16 | Accuracy Maps. *Visualisation of normalised accuracy maps on the behavioural dataset for all 16 categories averaged across human participants (n=30) and averaged across model seeds (n=5) for CNNs, LCNs, All-TNNs (α = [1, 10, 100], SimCLR) and TDANN (1 seed for the supervised version and 5 seeds for the self-supervised version).*

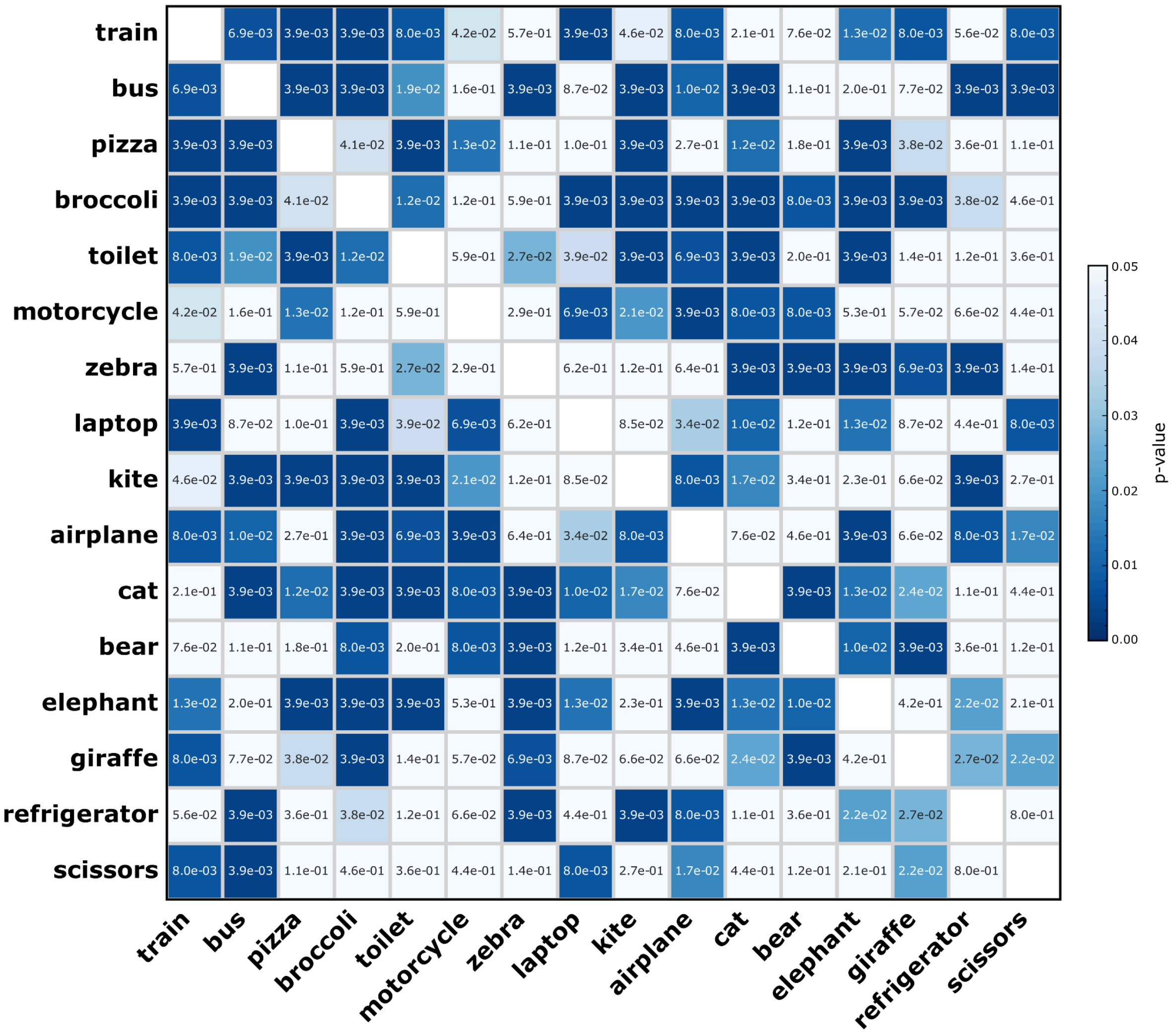

Figure S17 | Human accuracy maps reveal object-specific variation. *We test the individual cells of the ADM, representing the dissimilarity of category-pairs in the average spatial accuracy maps, against a null-distribution obtained by permuting the respective participant-based category maps. FWER correction is performed via BH-FDR correction with p<0.05.*

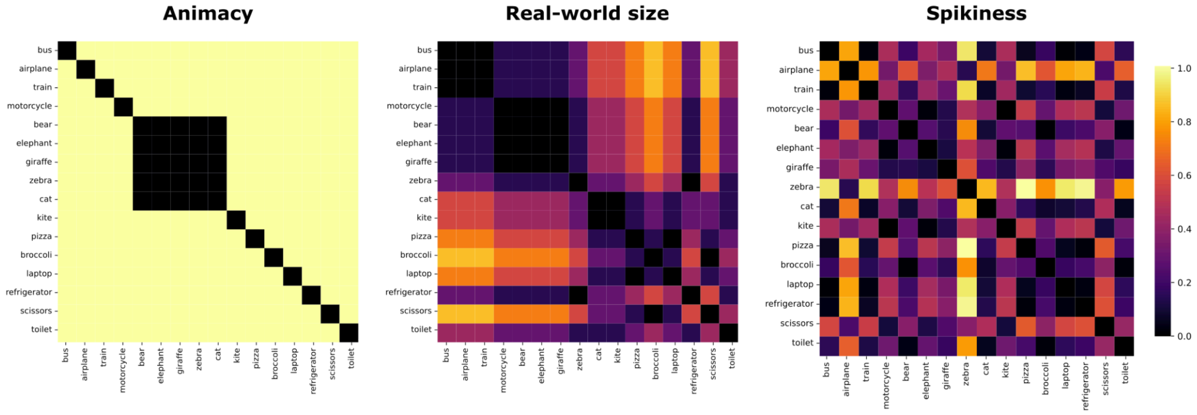

Figure S18 | ADM components used as predictors for hierarchical GLM modelling. *The non-negative least squares GLM on the average human ADM was performed using 3 predictor distance matrices: animacy, real-world size and spikiness (see Methods for calculation of the latter two).*

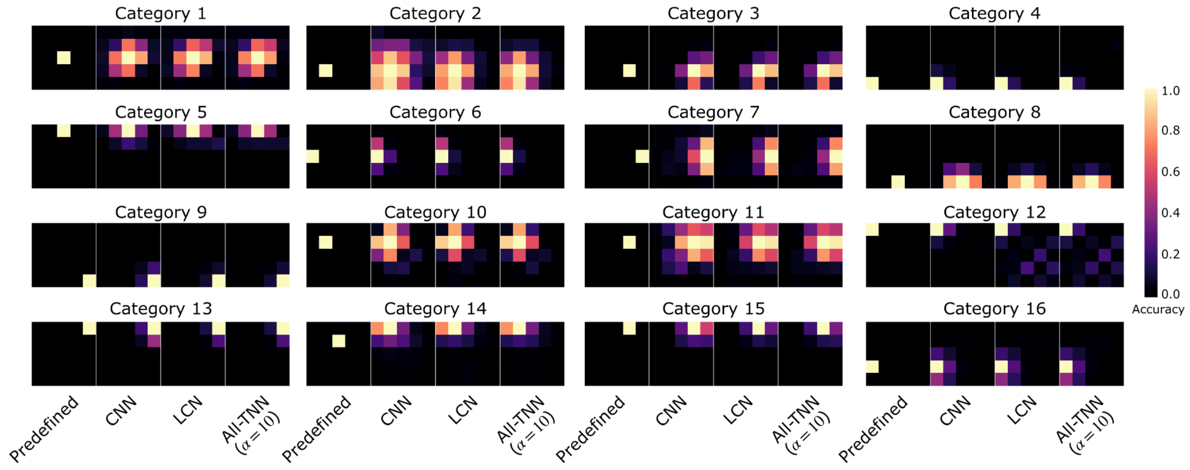

Figure S19 | Training models with a predefined spatial prior. *To investigate the extent to which all models can learn spatial biases, we create a novel toy dataset in which we place COCO segmentations on natural backgrounds on a fixed location (see Methods). After training the networks on the fixed spatial location dataset, we find that CNNs, LCNs and All-TNNs (α=10) reflect the spatial prior in their accuracy maps. This shows that all model types are able in principle to capture spatial biases in this simplified scenario. However, CNNs tend to mirror these biases in a slightly more dispersed manner compared to LCNs and All-TNNs (α=10) in most cases.*

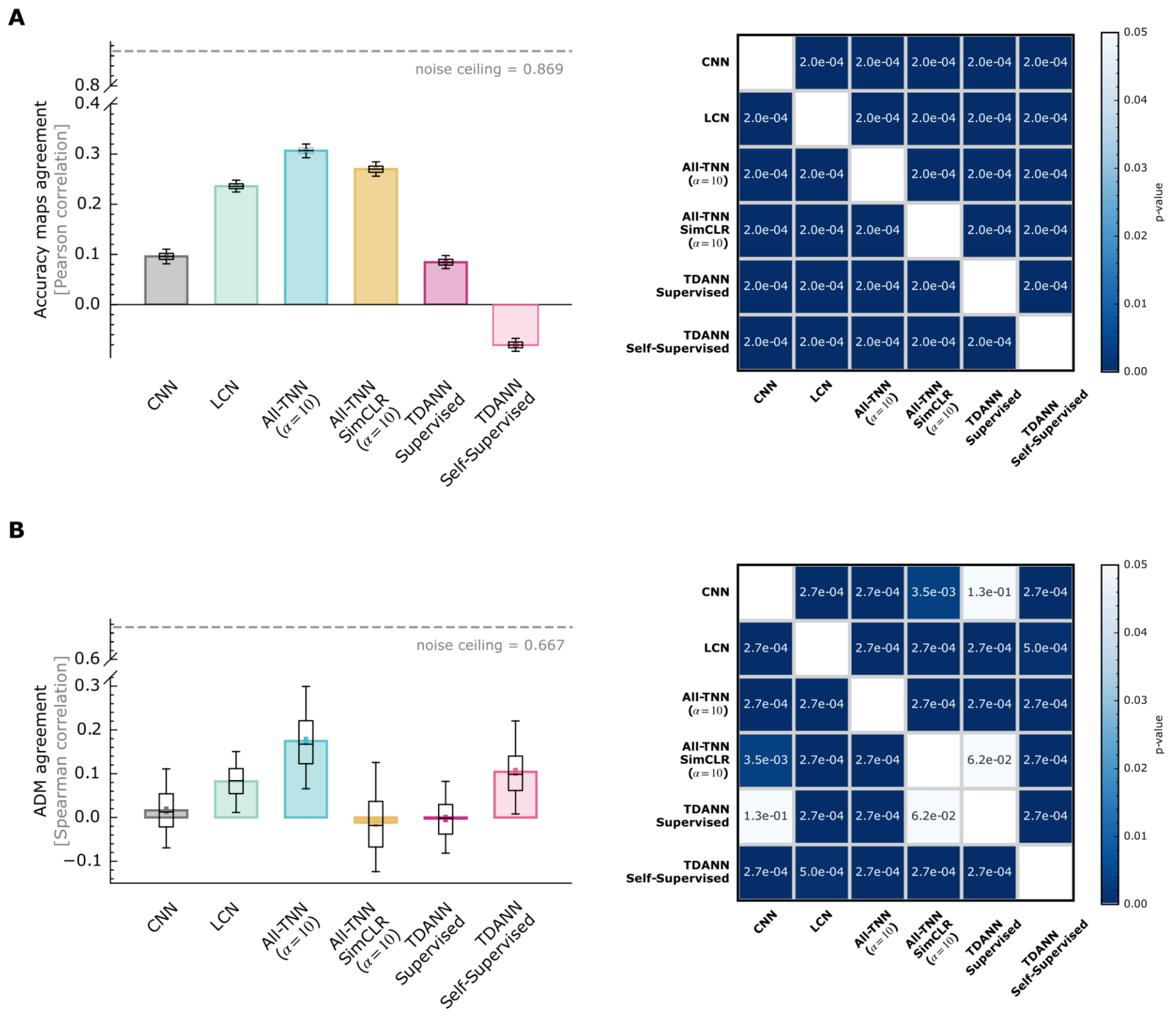

Figure S20 | Testing SimCLR-trained All-TNN and TDANN on behavioural agreement with human spatial biases. ***A.*** *Left: Both supervised and self-supervised All-TNNs reflect positional dependencies of the average human accuracy maps significantly better than both supervised and self-supervised TDANN (see Methods, 1 seed for the TDANN supervised model, 5 seeds for all other models). Noise ceiling calculated using split-half average human accuracy map correlation with Spearman-Brown correction. Right: BH-FDR-corrected p-values resulting from two-sided permutation tests (n=1e4) between model pairs.* ***B.*** *Left: Supervised All-TNNs significantly better capture category-specific spatial biases (ADM agreement) in human behaviour compared to supervised and self-supervised TDANNs (see Methods, 1 seed for the TDANN supervised model, 5 seeds for all other models). Noise ceiling calculated using split-half average human ADM correlation with Spearman-Brown correction. Right: BH-FDR-corrected p-values resulting from two-sided permutation tests (n=1e4) between model pairs. For both A and B, data are presented as mean values with 95% confidence interval, boxplots show the data distribution, midline of the box represents the median, box limits indicate the 25th to the 75th percentile and whiskers extend to the 5th and 95th percentiles.*

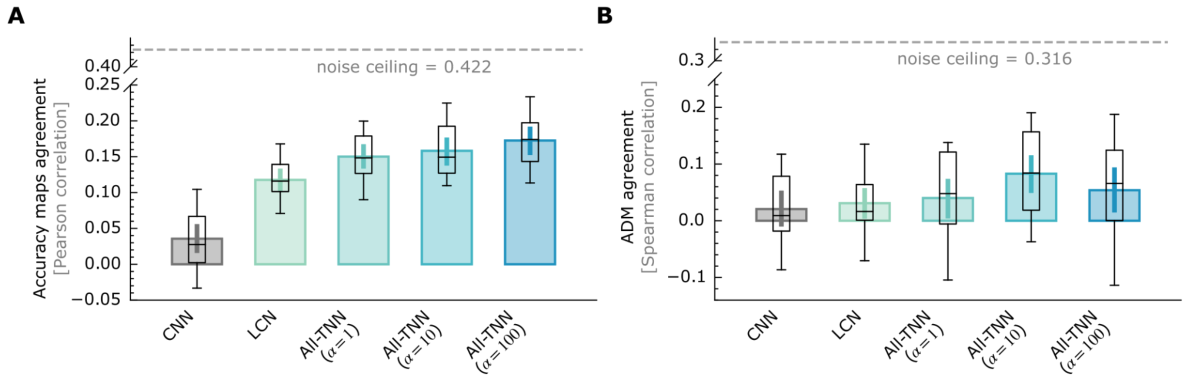

Figure S21 | Models better mirror spatial biases and category-specific variation in individual human visual behaviour. *In our main behavioural analyses (Fig. 4E and 4F), we measure agreement between models and average human behaviour. Here, we complementarily determine the agreement between models and individual human behaviour. The participant-level noise ceiling is the mean of the 30 correlations of individuals with the remaining 29 accuracy maps / ADMs (see Methods).* ***A.*** *All-TNNs significantly better align with the spatial biases of individual human behaviour than CNNs (measured using Pearson correlation between accuracy maps of model seeds and individuals; 30 participants, 5 seeds for each model; for statistical testing, see Supp. Fig. 22B, left).* ***B.*** *All-TNNs (α=10) capture categorical positional dependency of individual human behaviour significantly better than LCNs and CNNs (though other magnitudes of smoothness loss do not) (measured using Spearman correlation between model seed and individual ADMs; 30 participants, 5 seeds for each model; for statistical testing, see Supp. Fig. 22B, right). For both A and B, data are presented as mean values with 95% confidence interval, boxplots show the data distribution, midline of the box represents the median, box limits indicate the 25th to the 75th percentile and the whiskers extend to the 5th and 95th percentiles.*

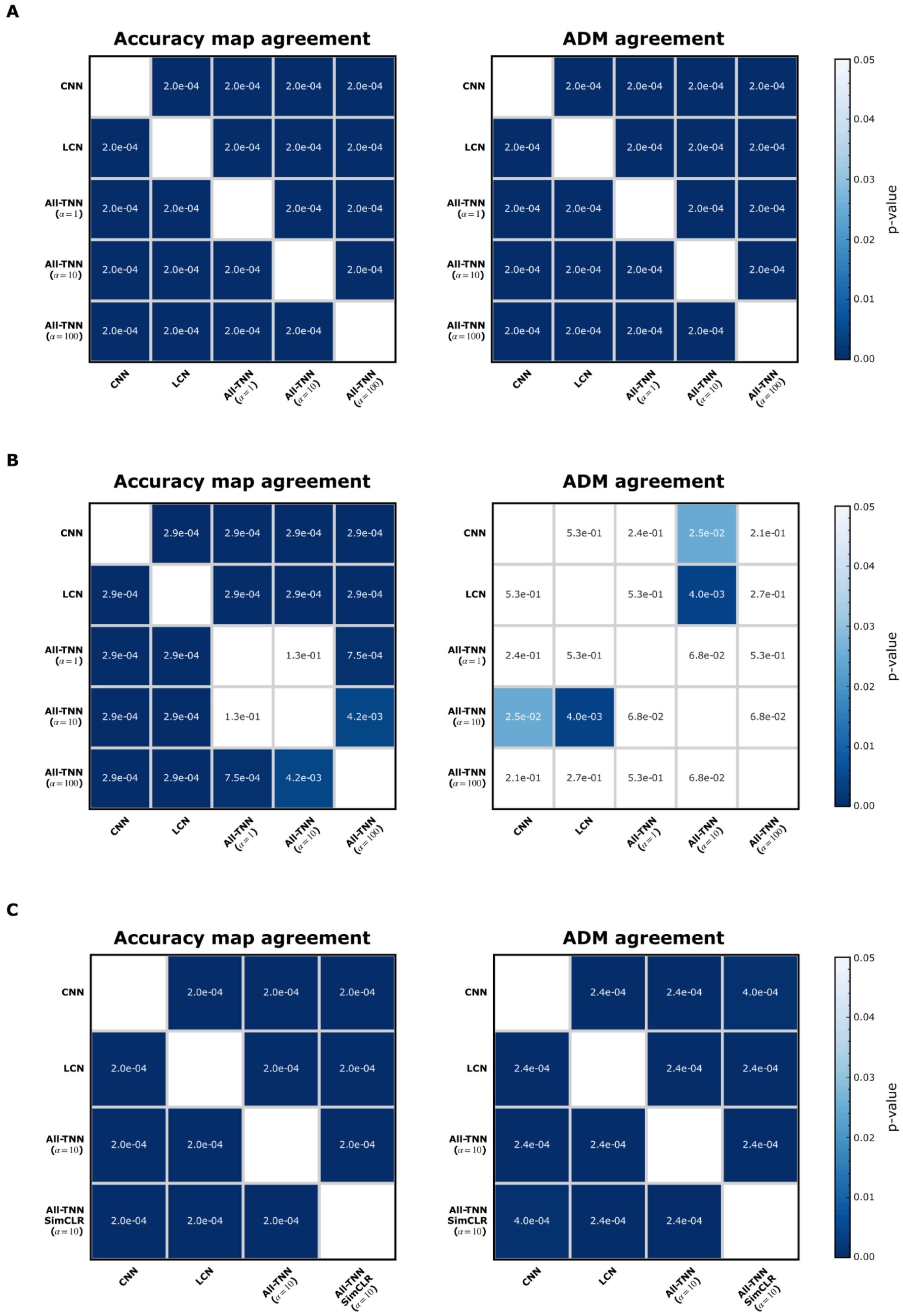

Figure S22 | Statistical tests for the analyses following the behavioural experiments. ***A.*** *Statistical test results for comparing All-TNNs, LCNs and CNN models with average human visual behaviour (Fig. 4E and Fig. 4F), BH-FDR-corrected p-values resulting from two-sided permutation tests (n=1e4) between model pairs.* ***B.*** *Statistical test results for All-TNNs, LCNs and CNN models with individual*

human behaviour (Supp. Fig. 21), BH-FDR-corrected p-values resulting from two-sided sign permutation tests (n=1e4) between model pairs. ***C.*** *Statistical test results for comparing SimCLR-trained All-TNNs, category-trained All-TNNs, LCNs and CNN models with average human visual behaviour (Fig. 5F and Fig. 5G), BH-FDR-corrected p-values resulting from two-sided permutation tests (n=1e4) between model pairs.*

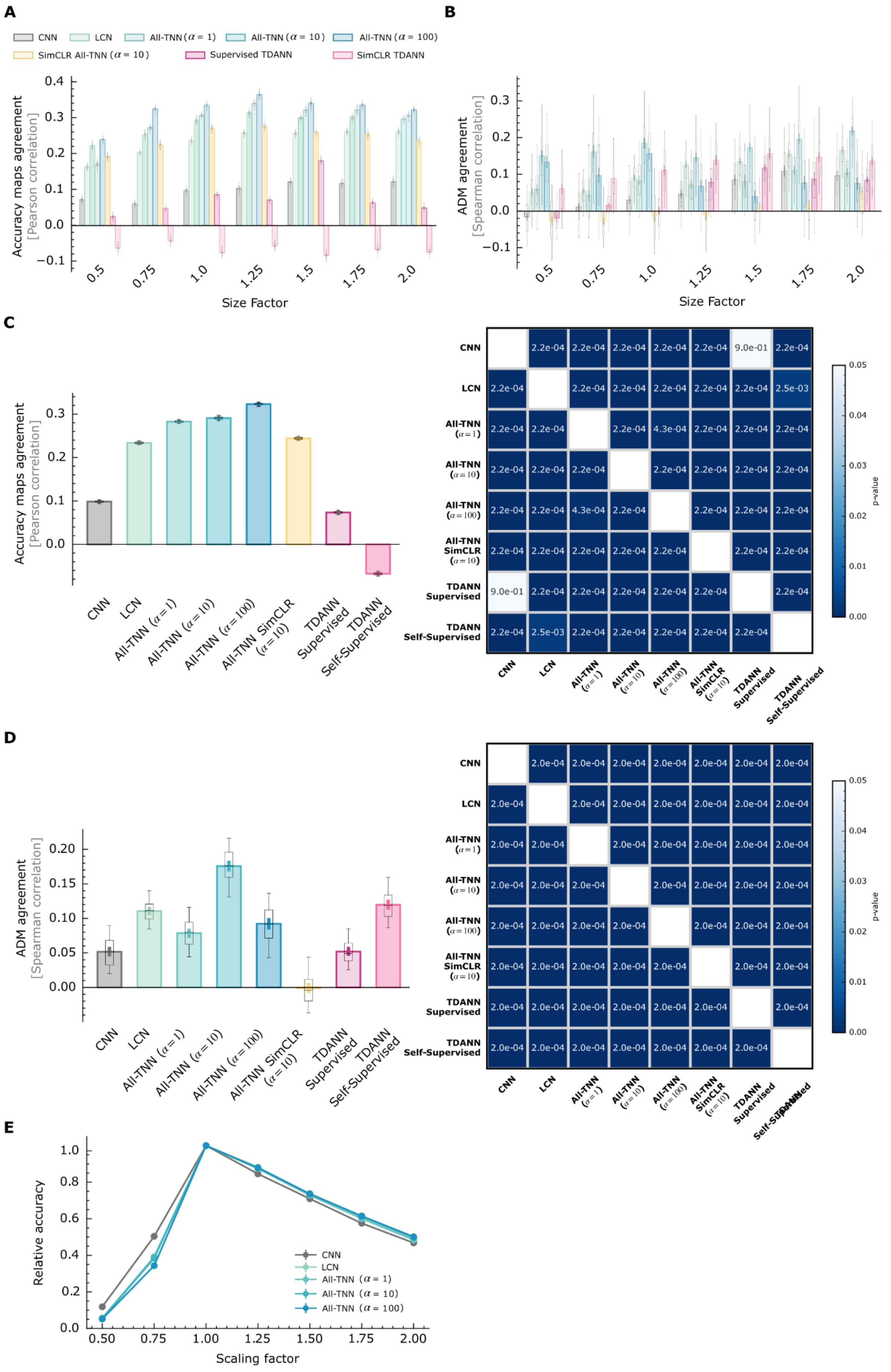
A
CNN
LCN
All-TNN (α = 1)
All-TNN (α = 10)
All-TNN (α = 100)
SimCLR All-TNN (α = 10)
Supervised TDANN
SimCLR TDANN
Accuracy maps agreement [Pearson correlation]
0.4 0.3 0.2 0.1 0.0 −0.1
0.5 0.75 1.0 1.25 1.5 1.75 2.0
Size Factor
B
ADM agreement [Spearman correlation]
0.3 0.2 0.1 0.0 −0.1
0.5 0.75 1.0 1.25 1.5 1.75 2.0
Size Factor
C
Accuracy maps agreement [Pearson correlation]
0.3 0.2 0.1 0.0
CNN
LCN
All-TNN (α=1)
All-TNN (α=10)
All-TNN (α=100)
All-TNN SimCLR (α=10)
TDANN Supervised
TDANN Self-Supervised
CNN: 2.2e-04 2.2e-04 2.2e-04 2.2e-04 2.2e-04 9.0e-01 2.2e-04
LCN: 2.2e-04 2.2e-04 2.2e-04 2.2e-04 2.2e-04 2.2e-04 2.5e-03
All-TNN (α=1): 2.2e-04 2.2e-04 2.2e-04 4.3e-04 2.2e-04 2.2e-04 2.2e-04
All-TNN (α=10): 2.2e-04 2.2e-04 2.2e-04 2.2e-04 2.2e-04 2.2e-04 2.2e-04
All-TNN (α=100): 2.2e-04 2.2e-04 4.3e-04 2.2e-04 2.2e-04 2.2e-04 2.2e-04
All-TNN SimCLR (α=10): 2.2e-04 2.2e-04 2.2e-04 2.2e-04 2.2e-04 2.2e-04 2.2e-04
TDANN Supervised: 9.0e-01 2.2e-04 2.2e-04 2.2e-04 2.2e-04 2.2e-04 2.2e-04
TDANN Self-Supervised: 2.2e-04 2.5e-03 2.2e-04 2.2e-04 2.2e-04 2.2e-04 2.2e-04
p-value
0.05 0.04 0.03 0.02 0.01 0.00
D
ADM agreement [Spearman correlation]
0.20 0.15 0.10 0.05 0.00
CNN
LCN
All-TNN (α=1)
All-TNN (α=10)
All-TNN (α=100)
All-TNN SimCLR (α=10)
TDANN Supervised
TDANN Self-Supervised
CNN: 2.0e-04 2.0e-04 2.0e-04 2.0e-04 2.0e-04 2.0e-04 2.0e-04
LCN: 2.0e-04 2.0e-04 2.0e-04 2.0e-04 2.0e-04 2.0e-04 2.0e-04
All-TNN (α=1): 2.0e-04 2.0e-04 2.0e-04 2.0e-04 2.0e-04 2.0e-04 2.0e-04
All-TNN (α=10): 2.0e-04 2.0e-04 2.0e-04 2.0e-04 2.0e-04 2.0e-04 2.0e-04
All-TNN (α=100): 2.0e-04 2.0e-04 2.0e-04 2.0e-04 2.0e-04 2.0e-04 2.0e-04
All-TNN SimCLR (α=10): 2.0e-04 2.0e-04 2.0e-04 2.0e-04 2.0e-04 2.0e-04 2.0e-04
TDANN Supervised: 2.0e-04 2.0e-04 2.0e-04 2.0e-04 2.0e-04 2.0e-04 2.0e-04
TDANN Self-Supervised: 2.0e-04 2.0e-04 2.0e-04 2.0e-04 2.0e-04 2.0e-04 2.0e-04
p-value
0.05 0.04 0.03 0.02 0.01 0.00
E
Relative accuracy
1.0 0.8 0.6 0.4 0.2 0.0
CNN
LCN
All-TNN (α = 1)
All-TNN (α = 10)
All-TNN (α = 100)
0.50 0.75 1.00 1.25 1.50 1.75 2.00
Scaling factor

Figure S23 | Effect of variation in object size on classification accuracy and behavioural agreement with human visual biases. *To investigate the effect of object size on the results of our behavioural experiment, we conduct a controlled experiment in which systematically vary the object size of the behavioural stimuli (see Methods).* ***A.*** *The finding that All-TNNs achieve higher accuracy map agreement with human accuracy maps than control models broadly extends to different object sizes (Pearson correlation; 30 participants, 1 seed for TDANN supervised model, 5 seeds for other models; data are presented as mean values with 95% confidence interval; see Methods).* ***B.*** *All-TNNs generally achieve higher ADM agreement with human ADMs than control models across different object sizes (Spearman correlation; 30 participants, 1 seed for TDANN supervised model, 5 seeds for other models; data are presented as mean values with 95% confidence interval; see Methods). We do observe size tolerance but not invariance: there is a small profile shift in tiny or large size factors****. C.*** *Averaging the accuracy map agreement across object size factors (see panel A), reveals that All-TNNs achieve significantly higher accuracy map agreement with averaged human accuracy maps than other control models across different object sizes (two-sided permutation test, n=1e4; BH-FDR-corrected).* ***D.*** *Averaging the ADM agreement across object size factors (see panel B), reveals that All-TNNs achieve significantly higher ADM agreement than other control models across different object sizes (two-sided permutation test, n=1e4; BH-FDR-corrected).* ***E.*** *Scaling the ecoset test set images with various factors and testing our models' classification accuracy on the respective scaled images shows that the relative accuracy changes similarly for all models across scaling factors. For figures A, B, C, and D, data are presented as mean values with 95% confidence interval, boxplots show the data distribution, midline of the box represents the median, box limits indicate the 25th to the 75th percentile and the whiskers extend to the 5th and 95th percentiles.*

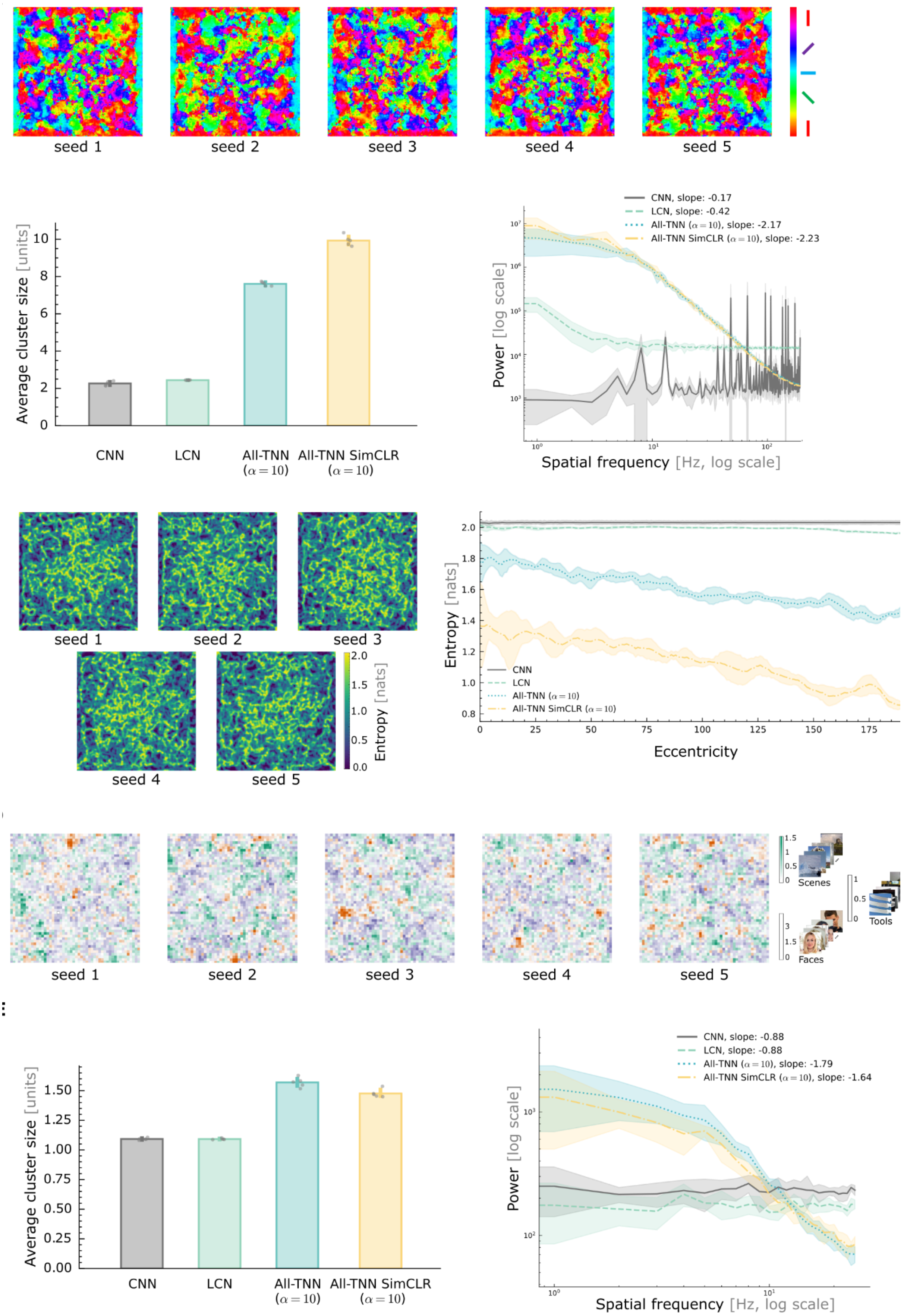

Figure S24 | Topography in All-TNNs trained with the self-supervised SimCLR training objective. *A.* *Orientation selectivity maps in the first layer of all seeds.* ***B.*** *Orientation selectivity maps (5 seeds for*

each model; data are presented as mean values with 95% confidence interval) of the first layer show clustering (left) and higher power in the low frequency spectrum (right). ***C.*** *Measuring the entropy of orientation selectivity maps in the first layer reveals an eccentricity-dependent increase in feature variety (data averaged across all seeds, shaded region indicates the 95% confidence interval).* ***D.*** *Category selectivity maps in the final layer of all seeds.* ***E.*** *Category selectivity maps do not show larger clusters than control models (left) but are smooth (right; 5 seeds for each model; both left and right data are presented as mean values with 95% confidence interval).*

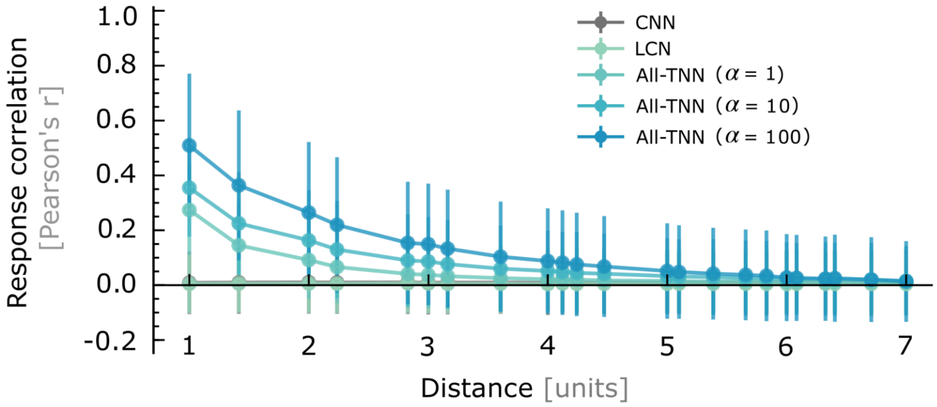

Figure S25 | Spatially correlated weight kernels in All-TNNs cause spatially correlated activities. *All-TNNs exhibit positive activity correlations across nearby units, which drop to orthogonality with larger distances, in line with biological findings (5 seeds for each model; data are presented as mean values with 95% confidence interval). This observation is not true for LCNs and CNNs. Data depicted for units in the final layer of each model.*

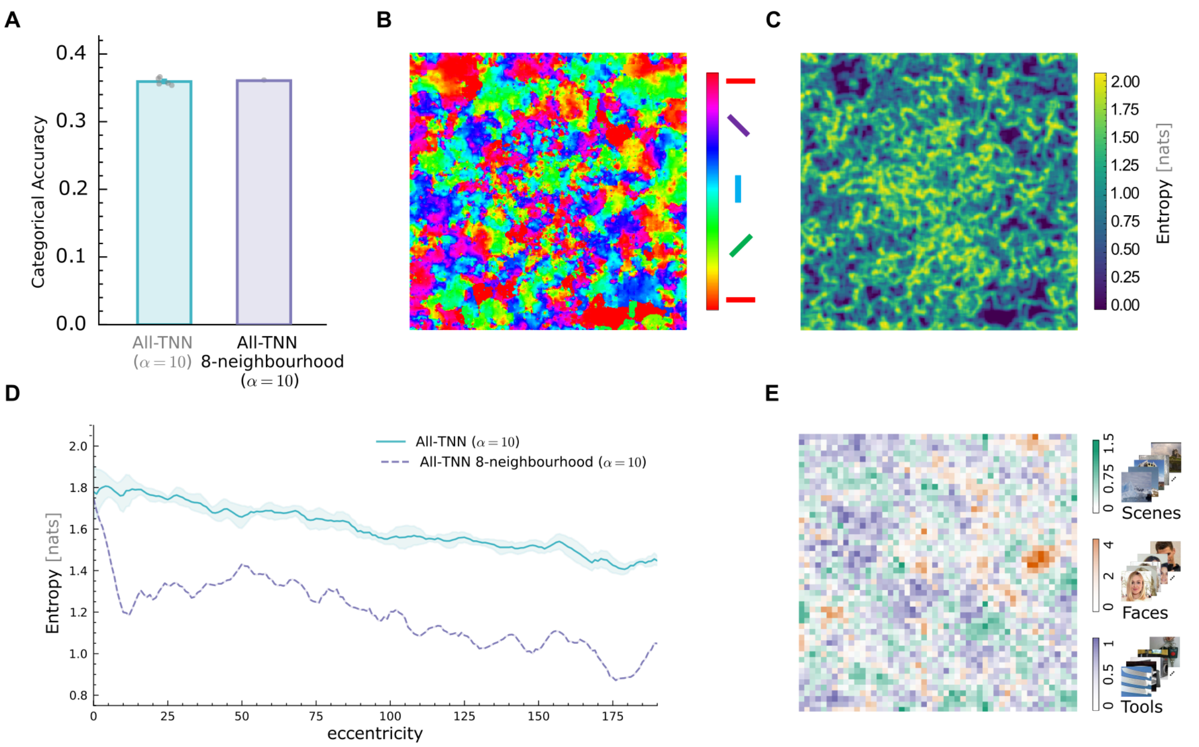

Figure S26 | All-TNN trained with smoothness loss calculated using the Moore neighbourhood. *To assure that the unit neighbourhood which the smoothness loss is calculated over is not too small, we expand the neighbourhood to include all 8 rather than 3 neighbours of a unit as in the main analyses. Training one such a network with* $\alpha = 10$ *results in comparable performance. The All-TNN with Moore neighbourhood (n=1) has a qualitatively similar topographical layout as the All-TNN (n=5; data are presented as mean values with 95% confidence interval) used in the main analyses, whereas smoothness and clustering are increased due to the stronger smoothness loss. Maps shown for the early stopping epoch (epoch 300).* ***A.*** *Classification performance on the test set of ecoset.* ***B.*** *Orientation selectivity in the first layer.* ***C.*** *Shannon entropy of orientation selectivity map.* ***D.*** *Shannon entropy of orientation selectivity map per eccentricity.* ***E.*** *Category selectivity of the final layer.*
