# Supplementary material for: End-to-end topographic networks as models of cortical map formation and human visual behaviour
Source: Nat Hum Behav. 2025 Jun 6;9(9):1975–91. doi: 10.1038/s41562-025-02220-7 (PMC12454150; doi:10.1038/s41562-025-02220-7)
Supplement: Supplementary file 2 — Reporting Summary [file 41562_2025_2220_MOESM2_ESM.pdf]

nature portfolio

Corresponding author(s): Tim C Kietzmann

Last updated by author(s): May 16, 2025

Reporting Summary

Nature Portfolio wishes to improve the reproducibility of the work that we publish. This form provides structure for consistency and transparency in reporting. For further information on Nature Portfolio policies, see our Editorial Policies and the Editorial Policy Checklist.

Statistics

For all statistical analyses, confirm that the following items are present in the figure legend, table legend, main text, or Methods section.

n/a	Confirmed	
☐	☒	The exact sample size (*n*) for each experimental group/condition, given as a discrete number and unit of measurement
☒	☐	A statement on whether measurements were taken from distinct samples or whether the same sample was measured repeatedly
☐	☒	The statistical test(s) used AND whether they are one- or two-sided<br>*Only common tests should be described solely by name; describe more complex techniques in the Methods section.*
☐	☒	A description of all covariates tested
☐	☒	A description of any assumptions or corrections, such as tests of normality and adjustment for multiple comparisons
☐	☒	A full description of the statistical parameters including central tendency (e.g. means) or other basic estimates (e.g. regression coefficient) AND variation (e.g. standard deviation) or associated estimates of uncertainty (e.g. confidence intervals)
☐	☒	For null hypothesis testing, the test statistic (e.g. *F*, *t*, *r*) with confidence intervals, effect sizes, degrees of freedom and *P* value noted<br>*Give P values as exact values whenever suitable.*
☒	☐	For Bayesian analysis, information on the choice of priors and Markov chain Monte Carlo settings
☒	☐	For hierarchical and complex designs, identification of the appropriate level for tests and full reporting of outcomes
☐	☒	Estimates of effect sizes (e.g. Cohen's *d*, Pearson's *r*), indicating how they were calculated

Our web collection on statistics for biologists contains articles on many of the points above.

Software and code

Policy information about availability of computer code

Data collection	Behavioural data collected with Matlab Psychtoolbox 3, ANNs trained with Python v.3.10 with Tensorflow v.2.12, and trained using NVIDIA H100 GPUs.
Data analysis	Python in-house code, Numpy (v.1.23.5) and Scikit-learn (v.1.4.0) packages. The code required to reproduce our results can be found on GitHub at https://github.com/KietzmannLab/All-TNN/.

For manuscripts utilizing custom algorithms or software that are central to the research but not yet described in published literature, software must be made available to editors and reviewers. We strongly encourage code deposition in a community repository (e.g. GitHub). See the Nature Portfolio guidelines for submitting code & software for further information.

Data

Policy information about availability of data

All manuscripts must include a data availability statement. This statement should provide the following information, where applicable:

- Accession codes, unique identifiers, or web links for publicly available datasets
- A description of any restrictions on data availability
- For clinical datasets or third party data, please ensure that the statement adheres to our policy

All analyses of human and model data were performed in custom Python software, making use of Tensorflow, Numpy, and Scikit-learn packages among others. The

data (model weights, selectivity datasets, behavioural study dataset and results) and code required to reproduce our results can be found on OSF (https://osf.io/6m3g4/) and Github (https://github.com/KietzmannLab/All-TNN/). Ecoset can be downloaded from https://huggingface.co/datasets/kietzmannlab/ecoset.

Research involving human participants, their data, or biological material

Policy information about studies with human participants or human data. See also policy information about sex, gender (identity/presentation), and sexual orientation and race, ethnicity and racism.

Reporting on sex and gender	We report the sex of our participants in text (17 female out of 30 participants).
Reporting on race, ethnicity, or other socially relevant groupings	N/A
Population characteristics	Participant age 21-30 years, mean=25.47 years, SD=2.5 years.
Recruitment	Participants were recruited via departmental student email lists at FU Berlin. As a result, self-selection biases are possible. We expect our results to generalize to similar student populations. All participants have normal or corrected-to-normal vision.
Ethics oversight	Ethics committee of the Department of Education and Psychology of the FU Berlin.

Note that full information on the approval of the study protocol must also be provided in the manuscript.

Field-specific reporting

Please select the one below that is the best fit for your research. If you are not sure, read the appropriate sections before making your selection.

☒ Life sciences ☐ Behavioural & social sciences ☐ Ecological, evolutionary & environmental sciences

For a reference copy of the document with all sections, see nature.com/documents/nr-reporting-summary-flat.pdf

Life sciences study design

All studies must disclose on these points even when the disclosure is negative.

Sample size	Our sample size (n=30) was selected based on running previous experimental paradigms, see e.g. Kaiser, Daniel, and Radoslaw M. Cichy. "Typical visual-field locations facilitate access to awareness for everyday objects." Cognition 180 (2018): 118–122. Given the extensive data collected per participant (2000 trials), our sample size is adequate to ensure reliable and generalizable findings.
Data exclusions	No participants or models were excluded from the analyses.
Replication	We trained multiple seeds per ANN model type (n=5 per model type) to ensure that the modelling results are not the result of random sampling error. All training models yielded consistent results.
Randomization	Stimulus sequence in the behavioural paradigm was randomised.
Blinding	Not applicable: stimulus presentation and scoring are fully computer-automated, responses are objective (correct/incorrect), and analysis is executed via a scripted pipeline. Experimenters have no influence on trial outcomes.

Reporting for specific materials, systems and methods

We require information from authors about some types of materials, experimental systems and methods used in many studies. Here, indicate whether each material, system or method listed is relevant to your study. If you are not sure if a list item applies to your research, read the appropriate section before selecting a response.

Materials & experimental systems

n/a	Involved in the study	
☒	☐	Antibodies
☒	☐	Eukaryotic cell lines
☒	☐	Palaeontology and archaeology
☒	☐	Animals and other organisms
☒	☐	Clinical data
☒	☐	Dual use research of concern
☒	☐	Plants

Methods

n/a	Involved in the study	
☒	☐	ChIP-seq
☒	☐	Flow cytometry
☒	☐	MRI-based neuroimaging
